# The Impact of Surface Roughness on Conformal Cooling Channels for Injection Molding

**DOI:** 10.3390/ma17112477

**Published:** 2024-05-21

**Authors:** Jan Hanzlik, Jiri Vanek, Vladimir Pata, Vojtech Senkerik, Martina Polaskova, Jan Kruzelak, Martin Bednarik

**Affiliations:** 1 Faculty of Technology, Tomas Bata University in Zlin, Vavreckova 5669, 760 01 Zlin, Czech Republic; j_hanzlik@utb.cz (J.H.); pata@utb.cz (V.P.); vsenkerik@utb.cz (V.S.); mpolaskova@utb.cz (M.P.); mbednarik@utb.cz (M.B.); 2 Faculty of Chemical and Food Technology, Slovak University of Technology in Bratislava, Radlinskeho 9, 812 37 Bratislava, Slovakia; jan.kruzelak@stuba.sk

**Keywords:** injection molding, conformal cooling channels, additive manufacturing, surface roughness, regression, ADAM, DMLS

## Abstract

Injection molding technology is widely utilized across various industries for its ability to fabricate complex-shaped components with exceptional dimensional accuracy. However, challenges related to injection quality often arise, necessitating innovative approaches for improvement. This study investigates the influence of surface roughness on the efficiency of conformal cooling channels produced using additive manufacturing technologies, specifically Direct Metal Laser Sintering (DMLS) and Atomic Diffusion Additive Manufacturing (ADAM). Through a combination of experimental measurements, including surface roughness analysis, scanning electron microscopy, and cooling system flow analysis, this study elucidates the impact of surface roughness on coolant flow dynamics and pressure distribution within the cooling channels. The results reveal significant differences in surface roughness between DMLS and ADAM technologies, with corresponding effects on coolant flow behavior. Following that fact, this study shows that when cooling channels’ surface roughness is lowered up to 90%, the reduction in coolant media pressure is lowered by 0.033 MPa. Regression models are developed to quantitatively describe the relationship between surface roughness and key parameters, such as coolant pressure, Reynolds number, and flow velocity. Practical implications for the optimization of injection molding cooling systems are discussed, highlighting the importance of informed decision making in technology selection and post-processing techniques. Overall, this research contributes to a deeper understanding of the role of surface roughness in injection molding processes and provides valuable insights for enhancing cooling system efficiency and product quality.

## 1. Introduction

Injection molding technology stands as one of the most pervasive and continually advancing methods for processing plastics. Its applications span various industrial sectors, notably, the automotive, aerospace, and defense industries. The technology’s primary advantage lies in its capability to fabricate complex-shaped components with exceptional dimensional accuracy, especially in high-series production. This encompasses the production of thin-walled and thick-walled components, as well as products with complex geometries. However, challenges concerning injection quality can arise during the production of certain components, necessitating attention [1,2,3].

Addressing such challenges often involves employing specialized injection molding methods, like multi-component injection molding or injection compression molding (ICM). On the other hand, when traditional injection molding must be used, high requirements on mold design are present, especially on the cooling system design, to ensure uniform temperature distribution across the product surface. Temperature uniformity plays a pivotal role in ensuring dimensional stability and minimizing residual stresses. However, conventional manufacturing methods may fall short in ensuring temperature uniformity for uniquely shaped products, prompting the adoption of conformal cooling facilitated by additive manufacturing technologies. Following that fact, the usage of conformal cooling in the injection molding design is still higher, but problems connected to an additive manufacturing technology, such as surface roughness, have not been extensively researched in this field [1,2,3,4].

Current research predominantly revolves around conformal cooling, with efforts focused on enhancing cooling system efficiency. Several studies by Venkatesh et al., Deepika et al., Jahan et al., Dimla et al., Park et al., and Shen et al. have compared conventional cooling methods with those employing additive manufacturing technologies, primarily through simulations due to the relatively high cost of additive manufacturing of steel parts [1,2,3,4,5,6]. As mentioned before, the conformal cooling application has the closest match with parts that require precise dimensional stability and quality such as optical parts [7,8,9,10,11,12,13,14,15].

However, additive manufacturing presents its own challenges, such as low-quality surfaces and high roughness, which can impede flow in cooling channels and hinder heat transfer, as mentioned by Han et al., Galati et al., and Babu et al. [16,17,18]. This fact is closely connected with the principle of additive manufacturing itself. For example, Direct Metal Laser Sintering (DMLS) creates the structure by sequentially depositing layers of microscopic grains and using high-power laser beams to selectively melt the powder grains in each layer. Among others, this type of technology is also used in the nuclear energy industry for its precision [19,20]. One of the main challenges to the acceptance of DMLS in conformal cooling design is the appearance of microscopic porosity defects in 3D-printed metal structures. Such pores are an artifact of the metal additive manufacturing process involving rapid melting and solidification without well-defined boundary conditions [21]. Depending on the shape, size, and orientation relative to structure surfaces, porosity defects could lead to material crack formation and cause structural failure [22,23,24]. Scanning electron microscopy (SEM) is a suitable method for determining how these defects could be detected [25].

As another option, ADAM technology has great application potential in this field and can be used in conformal cooling production. Another aspect that could be positive is that ADAM does not have as high safety requirements as DMLS technology. However, DMLS is still the most widely used technology in the field of conformal cooling, but ADAM technology could be a suitable alternative method of conformal cooling manufacturing. Nevertheless, surfaces of 3D-printed structures exhibit very low quality with high values of surface roughness either in the use of DMLS or ADAM technology [26,27].

DMLS technology is commonly used for manufacturing injection molding conformal cooling systems, and on the other hand, ADAM technology is not often used in this field but has great potential in this application. However, ADAM technology requires other additional steps after printing, such as washing out the binder. This can cause the creation of air capsules inside the part, which has a negative impact on the cooling effectivity. This fact is not in the scope of this study, and surely future research is necessary. Due to these aspects, this study is focused just on these two types of AM technologies and the surface roughness connected with these two types of AM technologies [26,27].

To optimize additive technologies for injection mold cooling, further processing of channel surfaces to reduce roughness is imperative, and these facts are discussed by Han et al., Günther et al., and Dumas et al. [28,29,30]. As conventional types of surface finishing are not suitable to use on internal surfaces, such as cooling channels, on the other hand, Abrasive Flow Machining (AFM) emerges as a suitable method for finishing internal channels produced using additive technologies [31,32,33,34]. However, AFM technology needs to be deeply researched in the field of injection molding.

Overall, the main task of this study is to verify the influence of the cooling channels’ surface roughness made by DMLS and ADAM technology on the total cooling systems’ effectivity and closely describe a possible benefit of the usage of finishing operations of the internal surfaces for the purpose of lowering surface roughness.

## 2. Materials and Methods

### 2.1. Overview of the Experiment

The experimental part is primarily focused on how surface roughness influences the efficiency of the conformal cooling channels. However, this experiment contains a roughness measurement of the test specimens made by additive technologies and also deals with a cooling channel flow analysis and evaluation of the measured data.

### 2.2. Materials and Equipment

The choice of which material can be used for the test specimens is limited by additive technology or the fact that the production of the injection mold cavities uses mainly tool steels. All these aspects had to be taken into mind and applied to the choice of material. Following the facts, the tool steel was chosen. The selected technologies and equipment for the fabrication of the test specimens are EOS M 290 (Direct Metal Laser Sintering—DMLS technology, EOS GmbH, Krailling, Germany) and Markforged Metal X (Atomic Diffusion Additive Manufacturing—ADAM technology, Markforged, Waltham, MA, USA).

Roughness measurements were performed using a Zygo Newview 8000 optical surface profiler (Lambda Photometrics, Harpenden, UK). However, for successful measurement, the test specimens had to be turned using the DMG MORI-NTX 1000 CNC machining center for turning and milling (DMG MORI USA, Hoffman Estates, IL, USA).

Scanning electron microscopy images were performed using Phenom XL G2 (Thermo Fisher Scientific, Waltham, MA, USA).

Cooling system flow analysis was performed using Moldex3D (version R14.0).

### 2.3. Test Specimens’ Design

Real injection mold cavities made by additive technology are relatively expensive in contrast to conventional manufacturing. Internal surface roughness measurement requires the destruction of the injection mold cavity. Due to this fact, the test specimens were designed. However, the idea of the test specimen design is inspired by the research of Han et al. [30]. These specimens substitute real injection mold cooling channels. Conformal cooling channels are made in these test specimens. The diameter of the cooling channel in this test specimen is 3 mm, and the channel has a helix pattern with a pitch of 10 mm.

**Figure 1:**
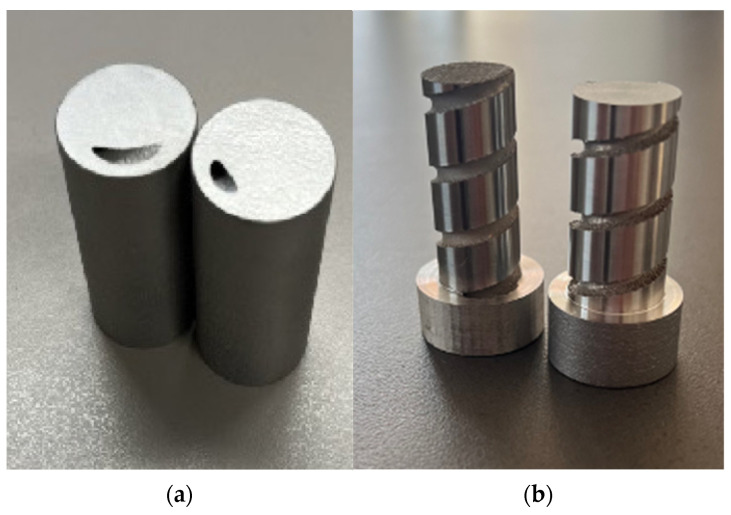
Test specimens made by DMLS technology: (**a**) fabricated test specimens (outer diameter is 20 mm and height of the specimen is 40 mm); (**b**) machined test specimens prepared for internal surface roughness measurement.

Machining of the test specimen is required for successful surface roughness measurement. Turning on a machining center was chosen as the appropriate method for this step. Machined test specimens are shown in Figure 1.

### 2.4. Internal Surface Roughness Measurement

As was mentioned before, one of the main goals of this research was to measure the internal surface roughness of the test specimens, simulating real conformal cooling channels. It was crucial to set a measurement procedure. First of all, fabricated test specimens had to be turned on the CNC machine because of exposure to the cooling channel inside of the test specimen. Next, the exposed cooling channel was scanned by an optical surface profiler along its length, and this means that each test specimen (specimen A—DMLS, specimen B—ADAM) was measured in five different places. Every one of the five measurements was translated from a curved surface to a flat surface and sliced up to fifty slices. Right after, parameters such as average roughness (Ra) and profile height (Rz) were evaluated. The Rz parameter is especially important for following cooling analyses. It is obvious that high peaks in the surface roughness affect the flow behavior of coolant media through the channel.

### 2.5. Scanning Electron Microscopy

Scanning electron microscopy could reveal porosity defects in the specimens. In this case, multiple images of the uncovered cooling channel of both specimens A and B have been taken. The voltage used during measurement was 10 kV.

### 2.6. Cooling System Flow Analysis

Performing the cooling system analysis was the next step in this research. It was important to closely simulate the process of the internal surface finishing because the Rz parameter of the finished surface roughness of the test specimens’ cooling channels is unknown. To achieve this, the default value of the Rz parameter was set as the value reached by the surface roughness measurement mentioned above. This default value was lowered by 10% until it reached a 90% smaller value of Rz. For each value of the Rz parameter, a single analysis was computed. Other process parameters were constant in all analyses.

Cooling channel analysis provides selected data, such as pressure along the cooling circuit or coolant media flow velocity.

It is important to mention that all the analyses were performed on the real injection molded optical part.

#### Analysis Parameters

Selected analysis parameters were constant in all performed analyses and are mentioned in Table 1 below.

## 3. Results

### 3.1. Internal Surface Roughness Measurement

The arithmetic mean of the Rz parameter was selected as a characteristic statistic parameter used for analysis. Its statistical significance was verified by estimating the coefficients of variation. Next, the estimated arithmetic means were recalculated to values of transformed arithmetic means using Box–Cox transformation, which is commonly used in engineering practice for this purpose [35].

The created surface scan can be interpreted as having a plane through the surface, which is interpreted as a zero in the scale on the right. However, this is a zero determined by statistical methods, namely, the least squares method. This means that all positive roughness values are scaled up to a maximum, which is colored red, while values below the plane are scaled in green and blue. A higher saturation of a given color or color combination then indicates a greater distance from the zero plane.

**Figure 2:**
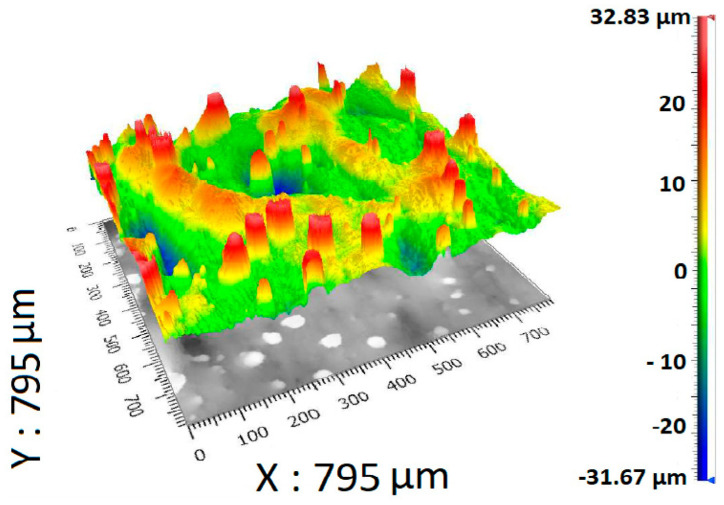
Projection of the measured data of specimen A containing color scale.

A projection of the measured data of specimen A is shown in Figure 2. The topology of the surface made by DMLS technology is shown in Figure 2, as well as values of the surface roughness.

Figure 2 shows the maximal and minimal measured values of surface roughness. In this case, the maximal value is 32.83 μm, and the minimal value is −31.67 μm. High peaks of the measured surface are colored red.

**Figure 3:**
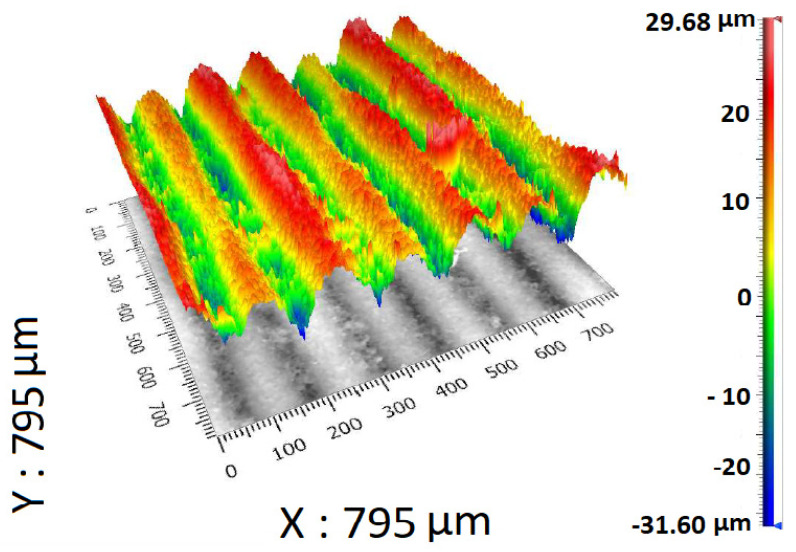
Projection of the measured data of specimen B containing color scale.

Figure 3 shows the maximal and minimal values of the measured surface. It is obvious that the maximal value is 29.68 μm and the minimal value is −31.60 μm. As was mentioned before, the topology of specimen B’s surface is dramatically different in comparison to the surface of specimen A. However, this type of surface shown in Figure 3 closely corresponds with ADAM technology.

**Figure 4:**
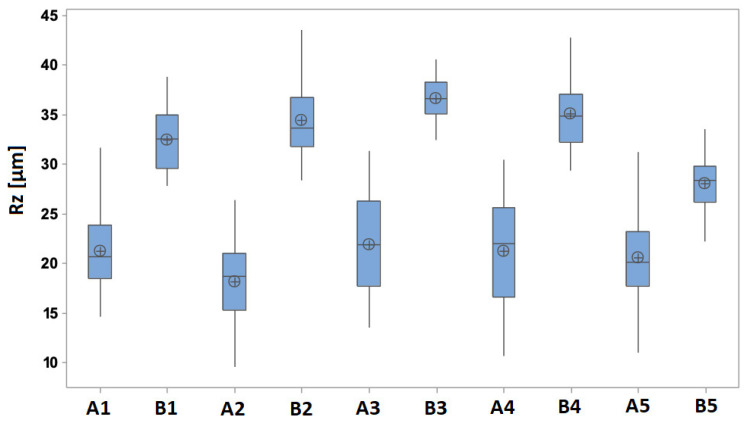
Comparison values of the Rz parameter of specimens A and B. A1–A5 and B1–B5 represent repeatability of the measurement.

Data in Figure 4 prove that the surface of specimen A and specimen B is topologically different, and this is caused by a type of additive technology. It is obvious that two topologically different surfaces are in the same general range of Rz and are not extremely different from each other.

Table 2 shows values of transformed arithmetic means of the Rz parameter, and these values are used during analyses as default values of the Rz parameter.

### 3.2. Scanning Electron Microscopy

A dense and uniform microstructure is a key to efficient cooling in injection mold inserts, as it ensures high thermal conductivity. Typically, a fine-grain structure with low porosity is preferred for optimal heat transfer. Incorporating complex geometries, like conformal cooling channels, can also improve cooling efficiency. The manufacturing process parameters are also critical in achieving the desired microstructure.

**Figure 5:**
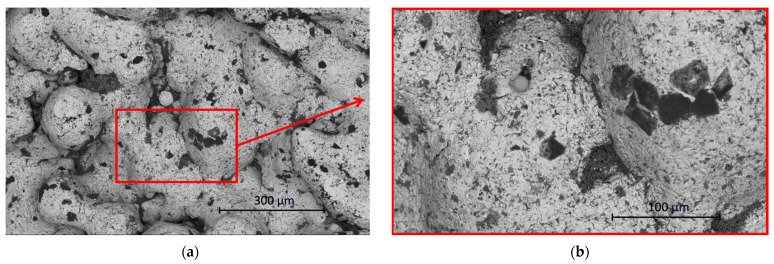
(**a**) SEM image of specimen A (magnification 500×); (**b**) detailed red rectangular area (magnification 1500×).

Results obtained by SEM match with the measurements of internal surface roughness measurements. Specimen A shows the type of surface that corresponds with a surface created by DMLS technology and does not show any significant type of porosity defect. This type of surface can be seen in Figure 5. This type of AM technology shows that surface structure can contain unmelted particles of the metal powder. This fact also can be seen in Figure 5.

**Figure 6:**
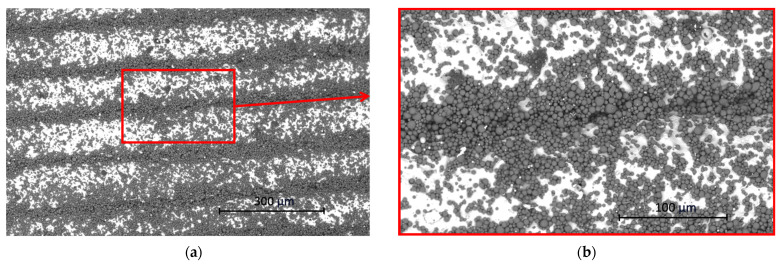
(**a**) SEM image of specimen B (magnification 500×); (**b**) detailed red rectangular area (magnification 1500×).

Figure 6 shows the surface of specimen B made by ADAM technology. As can be seen, the surface is rapidly different in contrast with specimen A. SEM images of specimen B do not show any type of porosity, which could lead to coolant media leakage into the injection mold cavity. The surface structure of specimen B shows obvious signs of layers. This is caused by ADAM technology itself. The principle of ADAM technology is that parts are printed from filament and then sintered, and the principle is similar to FDM technology.

#### EDAX Analysis

The secondary output reached by SEM imaging was the elemental composition of the specimens. As can be seen in Table 3, the elemental composition of specimen A qualitatively corresponds with tool steel 1.2709, which is the tool steel used for specimen A fabrication. On the other hand, Table 4. shows the elemental composition of specimen B, which qualitatively matches tool steel 1.2344. This type of tool steel was used for specimen B fabrication.

### 3.3. Cooling System Flow Analysis

Coolant pressure, Reynolds number, and streamline total velocity are the parameters that were evaluated. The surface roughness of the cooling channel influences all these parameters mentioned above. The visualization of the mentioned parameters is shown in the figures below, and values of every parameter for each Rz parameter are summarized in Table 5 and Table 6.

**Figure 7:**
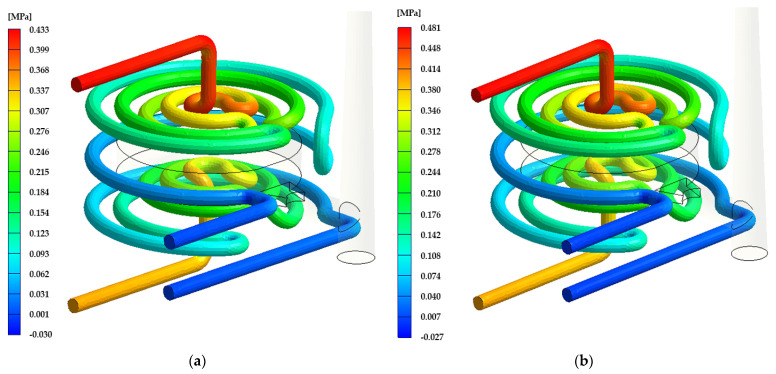
Visual coolant pressure interpretation: (**a**) DMLS technology (Rz = 21 μm); (**b**) ADAM technology (Rz = 34 μm).

As can be seen in Figure 7, the maximal value of the coolant pressure is 0.481 MPa for Rz = 34 μm. This value decreases with the channel length from the inlet to the outlet of the cooling circuit. This maximal pressure value in the cooling channel defines the minimal performance of the cooling unit. Such a high surface roughness of the channels creates flow resistance of the coolant media, and the pressure is then higher.

**Figure 8:**
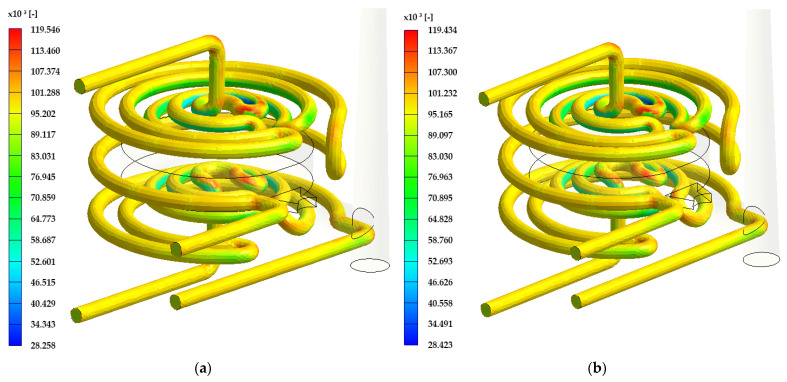
Visual Reynolds number interpretation: (**a**) DMLS technology (Rz = 21 μm); (**b**) ADAM technology (Rz = 34 μm).

Figure 8 shows the Reynolds number throughout the channel length. The maximum value in the case of Rz 34 μm is 119.43 × 10^3^, and the average value of the Reynolds nr. is 95,810. As can be seen, most of the channel is covered by yellow color, which matches with an average value.

**Figure 9:**
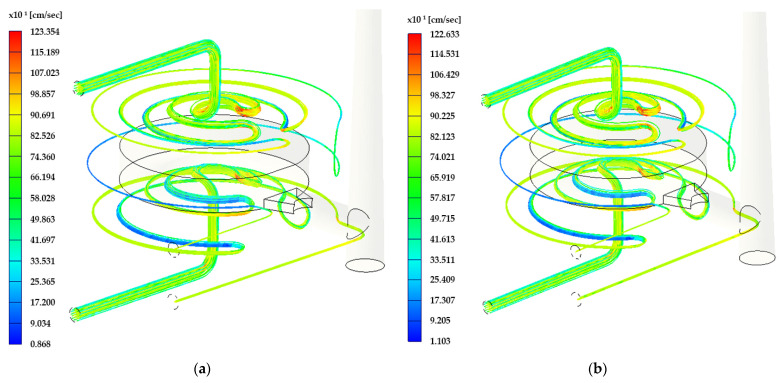
Visual flow velocity interpretation: (**a**) DMLS technology (Rz = 21 μm); (**b**) ADAM technology (Rz = 34 μm).

Flow velocity streamlines are shown in Figure 9 for the roughness parameter Rz = 34 μm and Rz = 21 μm. It is obvious that the maximal value of flow velocity is in the corners of the cooling channel and is equal to 1226.33 cm/s in the case of Rz = 34 μm. 

Table 5 summarizes the computed data of all parameters mentioned above. The maximal value of the Rz parameter for DMLS technology is 21 μm and the minimal value of Rz is 2.1 μm, which is a value obtained by simulating the cooling channel finishing process, and this value is commonly reachable by conventional drilling. Coolant pressure decreases by lowering Rz. On the other hand, Reynolds nr. and flow velocity increase when Rz decreases.

Table 6 summarizes the computed data of all parameters mentioned above. The maximal value of the Rz parameter is slightly higher for ADAM technology than DMLS. The minimal value of Rz is 90% lower than the maximal value. However, coolant pressure has a decreasing pattern from the highest to the lowest Rz. Flow velocity and Reynolds nr. grow with decreasing Rz.

The simulation software used for the analyses only allows for defining channel roughness in micrometers. It does not account for other variables from DMLS and ADAM, such as unique surface characteristics and internal morphology. This limitation might explain why the studied parameters seem similar between the two technologies, despite significant differences in their surface roughness and structure.

#### Regression Models

Based on the collected data, appropriate regression models were formulated to elucidate the impact of surface roughness on the observed characteristics. These models were developed using statistical software, such as Minitab^®^ 2017 (version 17.1, Minitab Inc., State College, PA, USA) and QC-Expert 3.3 (TriloByte, Staré Hradiště, Czech Republic).

The formulated model takes the following form:

(1) $$y=b_{0}+b_{1}x+b_{2}x^{2}+b_{3}x^{3}$$

where y represents the observed characteristic, x denotes surface roughness (μm), and b_0_, b_1_, b_2_, and b_3_ are the estimates of regression parameters.

During the process of model selection, rigorous tests for statistical significance were conducted. The outcome of these tests led to the rejection of the hypothesis of insignificance, indicating the robustness of the chosen regression model. Additionally, a calculation of the predicted correlation coefficient and median quadratic error of prediction was performed with the intent to estimate the regression parameters and find the type of regression function on a confidential level of 1 − α = 0.95, i.e., α = 0.05. Following data processing, a regression triplet was tested [36].

**Figure 10:**
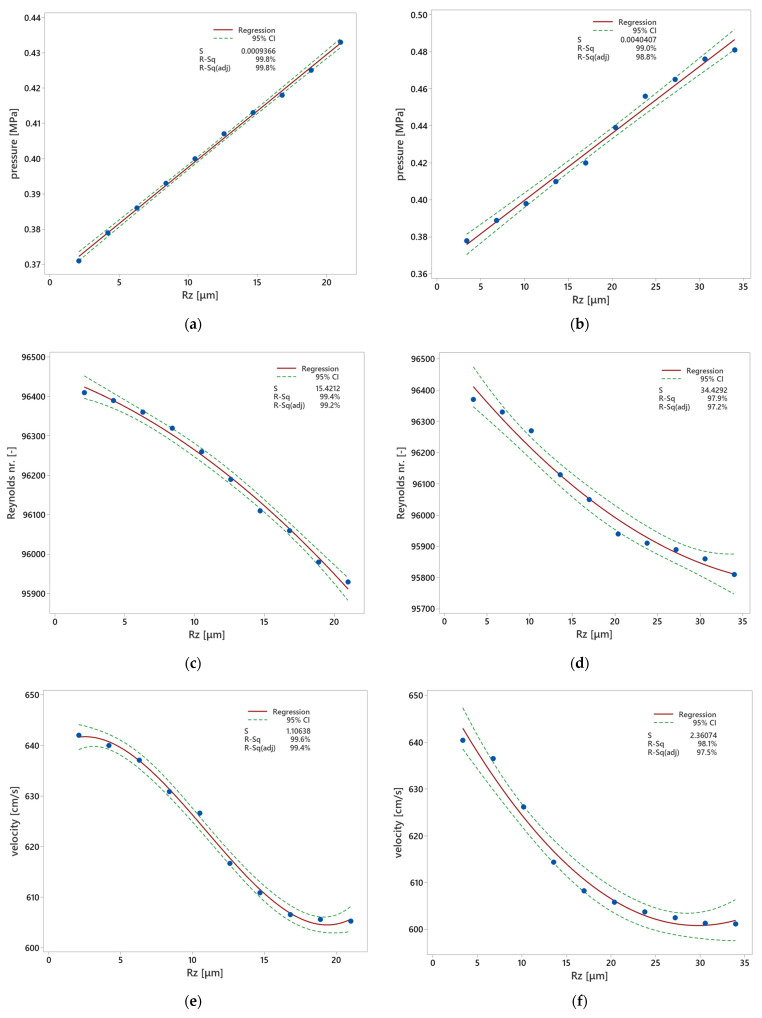
Proposed regression models—the influence of surface roughness on coolant pressure, Reynolds nr., and flow velocity in the cooling channels: (**a**) DMLS technology pressure; (**b**) ADAM technology pressure; (**c**) DMLS technology Reynolds nr.; (**d**) ADAM technology Reynolds nr.; (**e**) DMLS technology flow velocity; (**f**) ADAM technology flow velocity.

The results shown in Figure 10, Table 7 and Table 8 demonstrate that surface roughness influences coolant pressure, Reynolds nr., and flow velocity. Linear behavior of the regression model appears in the case of the pressure for both technologies. When surface roughness is lower, the pressure linearly decreases. On the other hand, when surface roughness decreases, both Reynolds nr. and flow velocity nonlinearly grow. This fact is positive, in contrast with the performance of the injection molding cooling systems.

## 4. Discussion

This study investigated the impact of surface roughness on the efficiency of conformal cooling channels produced using additive manufacturing technologies. The results of the experiment shed light on the intricate relationship between surface roughness, coolant flow dynamics, and pressure within the cooling channels, providing insights crucial for optimizing injection molding processes.

The experiment compared two additive manufacturing technologies, Direct Metal Laser Sintering (DMLS) and Atomic Diffusion Additive Manufacturing (ADAM), in terms of surface roughness. It was found that the surface roughness values differed significantly between the two technologies, with DMLS exhibiting lower roughness compared to ADAM. This discrepancy can be attributed to the inherent differences in the manufacturing processes of these technologies, particularly the deposition and melting of metal powders. These findings correspond with recent studies in this area by Galati et al. and Babu et al. [26,27].

Surface roughness plays a critical role in influencing coolant flow dynamics within the cooling channels. Higher surface roughness results in increased flow resistance, leading to higher coolant pressures within the channels. This finding was consistent across both DMLS and ADAM technologies. The visualization of coolant pressure distribution along the channels highlighted the importance of surface smoothness in achieving uniform flow and pressure distribution, especially in complex geometries. The mentioned finding could bring improvement in the research by Venkatesh et al. and Dimla et al. [1,4].

Regression models were developed to quantitatively describe the relationship between surface roughness and key parameters, such as coolant pressure, Reynolds number, and flow velocity. These models provided valuable insights into how variations in surface roughness affect coolant flow behavior. It was observed that while coolant pressure exhibited a linear decrease with decreasing surface roughness, Reynolds number and flow velocity showed nonlinear increases. This suggests that optimizing surface roughness can lead to more efficient coolant flow and enhanced cooling performance in injection molding processes.

The findings of this study have significant practical implications for the design and optimization of injection molding cooling systems. By understanding the intricate relationship between surface roughness and coolant flow dynamics, manufacturers can make informed decisions regarding the selection of additive manufacturing technologies and post-processing techniques to achieve desired surface quality and cooling efficiency. Additionally, the developed regression models provide a valuable tool for predicting cooling system performance based on surface roughness parameters, facilitating the optimization of injection molding processes for enhanced productivity and product quality.

In the end, the main target of this study was quantifying the influence of conformal cooling channels’ surface roughness. The designed regression models should contribute to better prediction of the cooling channels’ finishing process influence on the effectivity of the whole injection mold cooling systems and also on the overall quality of the injection molded parts.

## 5. Conclusions

In conclusion, this study represents the importance of surface roughness in influencing the efficiency of conformal cooling channels in injection molding processes. By clarifying the complex interplay between surface roughness, coolant flow dynamics, and pressure distribution, the research provides valuable insights for optimizing cooling system design and enhancing the overall productivity and quality of injection molded components. Further research in this area should focus on exploring advanced post-processing techniques to further refine surface roughness and optimize cooling system performance in real practice and focus on to how non-destructively measure and evaluate surface roughness inside the cooling channels, especially using computer tomography equipment.

## Figures and Tables

**Table 1:** Selected analysis parameters.

Parameter	
Channel diameter	4 mm
Coolant medium	Water (90 °C)
Defined flow rate	6 L/min
Mesh type	Volumetric
Element type	Tetra
Number of elements	172,374
Bad element	0
Solver	Standard
Analysis type	3D solid cooling channel
Turbulence modeling	Yes
Cooling channel mesh aspect ratio range	0.7–1

**Table 2:** Values of transformed arithmetic means of the Rz parameter.

Parameter	Specimen A (DMLS)	Specimen B (ADAM)
Rz [μm]	21	34

**Table 3:** EDAX analysis of specimen A.

Element	Atomic Concentration [%]	Weight Concentration [%]
C	31.65	11.01
O	20.08	9.31
Al	2.31	1.80
Si	2.09	1.70
Ca	2.50	2.90
Fe	29.63	47.95
Ni	10.23	21.12
Mo	1.51	4.20

**Table 4:** EDAX analysis of specimen B.

Element	Atomic Concentration [%]	Weight Concentration [%]
C	10.69	5.39
O	41.33	27.77
Al	36.95	41.86
V	2.19	4.70
Cr	2.74	5.99
Fe	6.09	14.29

**Table 5:** Analysis results summary for DMLS technology.

Rz [μm]	Pressure [MPa]	Reynolds nr. [-]	Flow Velocity [cm/s]
21	0.433	95,930	605.3
18.9	0.425	95,980	605.6
16.8	0.418	96,060	606.6
14.7	0.413	96,110	610.9
12.6	0.407	96,190	616.7
10.5	0.400	96,260	626.6
8.4	0.393	96,320	630.9
6.3	0.386	96,360	637.1
4.2	0.379	96,390	640.0
2.1	0.371	96,410	642.0

**Table 6:** Analysis results summary for ADAM technology.

Rz [μm]	Pressure [MPa]	Reynolds nr. [-]	Flow Velocity [cm/s]
34	0.481	95,810	601.2
30.6	0.476	95,860	601.3
27.2	0.465	95,890	602.5
23.8	0.456	95,910	603.8
20.4	0.439	95,940	605.8
17	0.420	96,050	608.3
13.6	0.410	96,130	614.4
10.2	0.398	96,270	626.2
6.8	0.389	96,330	636.5
3.4	0.378	96,370	640.4

**Table 7:** Estimations of the regression parameters—pressure and flow properties.

Type of AM Technology	Tested Parameter	Estimations of Regression Parameters			
		b_0_	b_1_	b_2_	b_3_
DMLS	Pressure [MPa]	3.656 × 10^−1^	3.195 × 10^−3^	-	-
	Reynolds nr. [-]	9.645 × 10^4^	−1.262 × 10^1^	−6.270 × 10^−1^	-
	Flow velocity [cm/s]	9.640 × 10^4^	1.153 × 10^1^	–3.120 × 10^0^	7.194 × 10^−2^
ADAM	Pressure [MPa]	3.673 × 10^−1^	3.472 × 10^−3^	-	-
	Reynolds nr. [-]	9.652 × 10^4^	−3.456 × 10^1^	3.998 × 10^–1^	-
	Flow velocity [cm/s]	6.546 × 10^2^	−3.614 × 10^0^	6.078 × 10^–2^	-

**Table 8:** The characteristics of the designed regression models—pressure and flow properties.

Parameters	DMLS			ADAM		
	Pressure	Reynolds nr.	Flow Velocity	Pressure	Reynolds nr.	Flow Velocity
Coefficient of Multiple Correlation	9.989 × 10^−1^	9.969 × 10^−1^	9.979 × 10^−1^	9.915 × 10^−1^	9.892 × 10^−1^	9.958 × 10^−1^
Coefficient of Determination	9.979 × 10^−1^	9.938 × 10^−1^	9.958 × 10^−1^	9.831 × 10^−1^	9.785 × 10^−1^	9.916 × 10^−1^
Predicted Correlation Coefficient	9.926 × 10^−1^	9.999 × 10^−1^	9.665 × 10^−1^	9.518 × 10^−1^	9.999 × 10^−1^	9.147 × 10^−1^
Mean Squared Error of Prediction	1.374 × 10^−6^	3.749 × 10^–1^	3.358 × 10^0^	3.071 × 10^−5^	1.242 × 10^0^	8.781 × 10^0^
Testing of Regression Triplet						
Fisher–Snedecor Test of Model Significance			Model is significant			
Scott’s Criteria of Multicollinearity			Model is correct			
Cook–Weisberg Score Test for Heteroskedasticity			Residue demonstrating homoskedasticity			
Jarque–Berra Test of Normality			Residue has a normal distribution			
Wald Test of Auto Correlation			Autocorrelation is insignificant			
Durbin–Watson Test of Auto Correlation			Negative autocorrelation of residues not demonstrated			

## Data Availability

Data are contained within the article.
